# Live Birth Rate in Patients With Premature Ovarian Insufficiency During Long-Term Follow-Up Under Hormone Replacement With or Without Ovarian Stimulation

**DOI:** 10.3389/fendo.2021.795724

**Published:** 2021-12-17

**Authors:** Bunpei Ishizuka, Masataka Furuya, Machiko Kimura, Eri Kamioka, Kazuhiro Kawamura

**Affiliations:** ^1^ Rose Ladies Clinic, Tokyo, Japan; ^2^ Department of Obstetrics and Gynecology, St. Marianna University School of Medicine, Kanagawa, Japan; ^3^ Department of Obstetrics and Gynecology, Advanced Reproductive Medicine Research Center, International University of Health and Welfare School of Medicine, Chiba, Japan

**Keywords:** premature ovarian insufficiency, infertility, live birth rate, pregnancy rate, ovarian stimulation

## Abstract

We analyzed data from 466 patients with premature ovarian insufficiency (POI) who wished to have a biological child and were followed up while undergoing hormone replacement (HR) therapy with or without ovarian stimulation (OS) between April 2014 and December 2020. OS was conducted in 6891 cycles in 429 patients (Group OS), whereas only HR (Group HR) was conducted in 1117 cycles in 37 patients. The follicle growth rate was 48.3% (207/429) per patient in Group OS and 5.4% (2/37) in Group HR (p<0.01). There were 51 live births (LBs) in 50 patients during follow-up. In Group OS, the LB rate was 5.8% (47/807) in cycles where *in vitro* fertilization (IVF) and embryo transfer were attempted (Group IVF), and 1.3% (3/236) in cycles where intrauterine insemination/timed intercourse was attempted (p<0.01). No pregnancies occurred in Group HR. Among the patients in Group IVF, the LB rate was significantly higher in patients aged <35 years at the initiation of follow-up than in patients who started at later ages (p<0.01). Among the cases who achieved an LB, 39 were patients with idiopathic POI (Group IVF-1, n=297) and seven were patients who had undergone surgical treatment for benign ovarian tumors (Group IVF-2, n=50); however, no LBs occurred in patients who had undergone treatment for malignancy (n=17), and only one in patients with chromosomal abnormalities (n=22). The LB rate per case in the patients in Group IVF-1 and those aged <35 years at the start of follow-up (Group IVF-1-a) was 24.1% (26/108), which was higher than those of the other age groups. The LB rate per case in the patients in Group IVF-1-a with <4 years of amenorrhea was 37.3% (19/51), and that in the patients in Group IVF-2 with <4 years of amenorrhea was 21.2% (7/33). These results suggest that infertility treatment is possible in some patients with POI, especially those that can be classified in Group IVF-1-a and Group IVF-2 with <4 years of amenorrhea. Therefore, OS combined with HR therapy should be considered for such patients before attempts at oocyte donation.

## Introduction

The incidence of premature ovarian insufficiency (POI), which refers to natural menopause before the age of 40 years, was originally reported to be 1% by Coulam (1). A recent national register study in Sweden reported that the incidence of idiopathic POI was 1.8%, and a meta-analysis reported that the global incidence was 3.7% (2, 3). Thus, POI is a somewhat common condition that may have ethnic, regional, and historical differences in terms of its incidence. Patients with POI could be infertile starting years before the onset of amenorrhea.

With the increasing trends of late marriages and childbearing in developed countries, the accumulated incidence of POI has increased among women who wish to conceive. In a study of 358 patients with idiopathic POI, the spontaneous remission of ovarian function, as indicated by the resumption of menstrual cycles and/or a decrease in follicle-stimulating hormone (FSH) levels to the normal range, occurred in 24% of all cases, and the spontaneous pregnancy rate in these patients after diagnosis was 4.4% (4). Furthermore, in observational studies of patients with POI who had received estrogen replacement therapy, the pregnancy rate was 4.8% (5). Attempts at ovulation induction in infertile patients with POI yielded an overall pregnancy rate of 6.3%, and controlled studies using a gonadotropin-releasing hormone agonist (GnRH-a) failed to show any difference in pregnancy rates compared with placebo (5–7). Therefore, after a diagnosis of POI, the spontaneous pregnancy rate is low and fertility treatment is considered to have little value. As a result, oocyte donation (OD) is considered to be the most reasonable treatment option for infertility in patients with POI (5, 8, 9). However, one study published in 1990 on ovulation induction with estrogen replacement and human menopausal gonadotropin (hMG) administration in patients with >12 months of amenorrhea reported a pregnancy rate per patient of 20% (19/91) (10). In the same study, it was reported that hMG stimulation with a GnRH-a (leuprolide acetate) without estrogen replacement therapy failed to produce any pregnancies in nine consecutive patients. Also, another study published in 2007 on patients with POI with >6 months of amenorrhea reported that hMG stimulation under estrogen replacement resulted in follicle growth and clinical pregnancy (CP) in 36% (9/25) and 16% (4/25) of the cases respectively, while no follicle growth was observed in patients to whom hMG was given without estrogen replacement (11). Therefore, ovarian stimulation (OS) by a short protocol of recombinant follicle-stimulating hormone (recFSH) and hMG (recFSH/hMG) combined with GnRH-a under estrogen replacement may be effective as infertility treatment in some patients with POI. However, to our knowledge, no large-scale retrospective cohort studies have been conducted on pregnancy and LB rates among patients with POI who wish to have their own biological child during long-term follow-up on hormone replacement (HR) therapy with and without OS with recFSH/hMG and GnRH-a. Given this background, the present study aimed to investigate CP and LB rates in 466 patients with POI who wished to conceive while under HR therapy with or without OS with recFSH/hMG and a GnRH-a.

## Materials and Methods

### Patients

This retrospective cohort study included 466 patients with POI who wished to conceive with their own gametes or receive oocyte cryopreservation for fertility preservation. Data were collected from patients who were first seen for infertility treatment at Rose Ladies Clinic in Tokyo, Japan from April 2014 to December 2017 and diagnosed as POI according to the European Society of Human Reproduction and Embryology (ESHRE) guidelines (12). Clinical data were collected until December 2020. The mean age ± standard error (SE) of the patients at the initiation of follow-up was 35.8 ± 0.2 years (median, 36.0; range, 22–46). The mean ± SE duration of amenorrhea of these patients at the initiation of follow-up was 6.0 ± 0.2 years (4.4; 0.3–26), and the mean ± SE duration of follow-up was 3.1 ± 0.1 years (3.1; 0.2–6.7). Of these 466 patients, 171 (36.7%, Group a) initiated the follow-up at our facility at <35 years of age, whereas 195 (41.8%, Group b) initiated the follow-up between 35–39 years of age and 100 (21.5%, Group c) initiated the follow-up at ≥40 years of age. Whether the patients were treated with recFSH/hMG stimulation under HR therapy or followed up with HR only depended on individual requests. According to the presence or absence of possible iatrogenic causes and chromosomal abnormalities, the patients were classified into the following four groups: patients with idiopathic POI (Group 1, n=360), patients after surgery for a benign ovarian tumor (Group 2, n=54), patients after treatment of malignancy (Group 3, n=22), and patients with chromosomal abnormalities (Group 4, n=30). Of the 466 patients, 429 underwent OS under HR therapy and 37 were observed with HR therapy only (Group HR). OS was conducted in 6891 cycles in 429 patients (Group OS), and HR only in 1117 cycles in 37 patients (Group HR). Among the patients in Group OS and Group HR, the mean age and duration of amenorrhea at the initiation of follow-up at our facility were 35.8 ± 0.2 and 6.0 ± 0.3 years and 35.4 ± 0.9 and 6.8 ± 0.9 years, respectively (n.s.). Among the 6891 cycles in 429 patients in Group OS, oocyte retrieval (OR) was attempted in 807 cycles in 201 patients (Group IVF), while intrauterine insemination (IUI)/timed intercourse (TI) was performed in 236 cycles in 68 patients (62 cases overlapped in both groups). The study protocol was approved by the Biomedical Ethics Committee of the Rose Ladies Clinic (RLC-19) and registered in the University Hospital Medical Association Network (UMIN 000040360).

### Inclusion and Exclusion Criteria

We retrospectively analyzed data on patients with POI who met the criteria of the ESHRE guidelines. The inclusion criteria were as follows: 1) patients with serum FSH >25 mIU/mL with hypogonadism defined as serum estradiol (E2) levels <20 pg/mL on at least two different occasions at least 4 weeks apart; 2) patients without spontaneous menstruation for >4 months based on self-reports; 3) patients whose last menstrual period occurred when they were <40 years of age; and 4) patients who wished to conceive using their own or cryopreserved oocytes. Patients we could not follow for more than one complete treatment cycle were excluded from the analysis.

### Hormone Replacement (HR) Therapy

After measuring circulating luteinizing hormone (LH), FSH, and E2 levels without any HR for at least 4 weeks, patients started taking daily oral conjugated estrogen (Premarin^®^; Pfizer, New York, NY, USA) at an initial dose of 0.625–1.875 mg (depending on body weight). We adjusted the dosage by measuring serum E2 levels to maintain 50–80 pg/mL. In cycles without OS, serum E2 levels were measured at the 7th to 14th day of the cycle before initiating the administration of medroxyprogesterone acetate (Provera^®^; Pfizer, Tokyo, Japan). When serum E2 levels exceeded 80–100 pg/mL, transvaginal ultrasonography was performed to confirm follicle development. When the follicle structure was observed on ultrasonography, we either observed spontaneous follicle growth or attempted OS with hMG (Folyrmon-P; Fuji Pharma Co., Ltd., Tokyo, Japan) or recFSH with or without a GnRH-a [cetrorelix acetate (Cetrotide^®^; Nippon Kagaku, Tokyo, Japan)] depending on serum LH and FSH levels. When follicles with a diameter >16–18 mm were observed, *in vitro* fertilization (IVF) combined with embryo transfer (ET) was attempted. When patients without any indications other than POI preferred to receive IUI/TI for financial reasons or because of their work schedule, IUI/TI was performed or instructed. When follicle development was not observed by day 14 of the cycle, medroxyprogesterone acetate (Provera^®^; Pfizer) 10–15 mg/day was administered for 10 days to induce withdrawal bleeding and start the next treatment cycle.

### Ovarian Stimulation (OS) Protocol

On days 3–5, after confirming that serum FSH and LH levels had decreased to within normal ranges (<11 mIU/mL), hMG (Folyrmon-P; Fuji Pharma Co., Ltd.) or recFSH (Gonal-f; Merck Seono Japan, Tokyo, Japan) administration was initiated with a starting dose of 225–450 IU/day (depending on body weight) together with GnRH-a [buserelin acetate (Buserelin); Fuji Pharma Co., Ltd.] at a starting dose of 450–1200 μg/day (depending on gonadotropin levels). Patients were seen at weekly intervals and serum E2, LH, and FSH levels were measured until signs of follicle growth were detected by serum E2 level and transvaginal ultrasound monitoring. We attempted to keep serum FSH levels >30 mIU/mL and LH levels <5 mIU/mL by adjusting the dosages of GnRH-a and recFSH/hMG. recFSH/hMG was discontinued when a dominant follicle >16 mm in diameter developed, serum E2 levels reached >150 pg/mL above basal levels at the start of the stimulation cycle, and oocyte maturation was triggered by 10,000 IU of human chorionic gonadotropin (hCG) administration (Gonadotropin; Aska Pharmaceutical Co., Ltd., Tokyo, Japan). OR was performed at 34–36 h after hCG injection. When patients did not desire to undergo IVF because of the above-mentioned reasons, IUI was performed at 24 h after hCG injection or TI was instructed. When signs of follicle growth were not seen after 4 weeks of treatment, recFSH/hMG administration was discontinued and medroxyprogesterone acetate (Provera^®^; Pfizer) 5–10 mg/day (depending on body weight and estrogen dosage) was administered for 10 days.

### *In Vitro* Fertilization (IVF)

When a mature oocyte was obtained during OR, conventional IVF or intracytoplasmic sperm injection (ICSI) was performed. ICSI was performed in cases with 1) no or poor fertilization in the previous cycles, 2) less than 0.8×10^6^ spermatozoa after preparation, and 3) sperm morphology <5% normal. Oocytes were retrieved by transvaginal ultrasound-guided aspiration at 36 h after hCG injection. Oocytes were cultured in ORIGIO Sequential Fert (CooperSurgical, Inc., Trumbull, CT, USA) in 6% CO_2_, 5% O_2_, and 89% N_2_ at 37°C until conventional IVF or ICSI. Oocytes were inseminated or injected with sperm using the standard ICSI technique at 3–5 h after OR. Oocytes were observed at 16–18 h after IVF or ICSI. Normal fertilization was confirmed by the presence of two pronuclei and extraction of the second polar body. Fertilized oocytes were cultured for 24 h in ORIGIO Sequential Cleav™ (CooperSurgical, Inc.) in 6% CO_2_, 5% O_2_, and 89% N_2_ at 37°C. The embryo grade was evaluated based on Veeck’s criteria (13). A good quality embryo was defined as one that had reached four cells and had <20% of its volume filled with fragment; these embryos were cryopreserved for later ET. When embryos did not fulfill the above criteria at day 2, we extended the culture until day 5. When these embryos were developed to the blastocyst stage and the blastocyst grade was evaluated according to Gardner’s criteria, only high-quality (AA, AB, BA, BB) and moderate-quality (AC, CA, BC, CB) blastocysts were cryopreserved. In most treatment cycles, OR was performed after more than a few weeks of OS and the endometrial condition was not ready for ET, so we employed a “freeze-all” strategy.

### Semen Evaluation and Preparation

Semen samples were collected *via* masturbation after 3–5 days of sexual abstinence and were liquefied for at least 30 min at room temperature. Sperm concentration and motility were assessed by microscopy using a Makler^®^ counting chamber (Sefi Medical Instruments, Haifa, Israel) according to World Health Organization criteria (14). The sperm preparation method for conventional IVF or ICSI was performed by a combination of density gradient centrifugation and the swim-up method as described below. Sperm samples were layered onto ORIGIO Gradient 90 (CooperSurgical, Inc.) and centrifuged at 500×g for 18 min. The supernatant and gradient medium just above the sperm pellet were removed and discarded. Then, the sperm pellet was washed by centrifugation at 200×g for 5 min in ORIGIO Sperm Wash (CooperSurgical, Inc.). The supernatant was then removed, and 0.5 mL sperm wash medium was gently layered on top of the sperm pellet to allow motile sperm to swim up. The tube was inclined at approximately 45° and incubated at room temperature for 15 min. After incubation, the supernatant was aspirated and transferred to a sterile tube. The sperm concentration and motility of the sperm suspension were then estimated. Finally, the sperm sample was stored at room temperature until conventional IVF or ICSI.

### Embryo Cryopreservation and Thawing

Embryo cryopreservation and thawing were performed using Cryotop methods, as described elsewhere (15). Embryo quality was assessed on day 2 according to Veeck’s criteria (13). High-grade embryos, which were defined as Grades 1–3 and those containing 4–6 blastomeres, were selected for vitrification. When the culture was extended to the blastocyst stage, high-quality (AA, AB, BA, BB) and moderate-quality (AC, CA, BC, CB) blastocysts according to Gardner’s criteria were cryopreserved. These embryos were then equilibrated in Equilibrium Solution (Kitazato Corp., Tokyo, Japan) at room temperature for 10 min. After equilibration, the embryos were transferred into Vitrification Solution (Kitazato Corp.), and then onto a Cryotop strip (Kitazato Corp.) with minimal Vitrification Solution and immediately submerged in liquid nitrogen. The vitrification step was completed within 1 min. Cryotop (Thawing Media; Kitazato Corp.) was subsequently capped with a straw and stored in storage tanks until thawing was complete.

### Embryo Transfer (ET)

Embryos were thawed on the day of ET. The vitrified embryo was warmed in Thawing Solution (Thawing Media; Kitazato Corp.) at 37°C for 1 min. After thawing, the embryo was placed into Diluent Solution (Thawing Media; Kitazato Corp.) for 3 min at room temperature to dilute the cryoprotectant, followed by stepwise washing in Washing Solution (Thawing Media; Kitazato Corp.) as follows: the first wash was performed for 5 min at room temperature, followed by a second wash for 1 min at room temperature. Embryos were then placed in Embryo Glue (Vitrolife AB; Västra Frölunda, Sweden) and cultured in a CO_2_ incubator at 37°C under 6% CO_2_ and 5% O_2_ until ET. Due to undetectable E2 levels in these patients, ET was performed under HR therapy using transdermal E2 (Estrana tape^®^; Hisamitsu Pharmaceutical, Tokyo, Japan) at a starting dose of 1.44 mg/48 h. The dose of transdermal E2 was increased every 48 h until serum E2 levels were >350 pg/mL and the endometrial thickness as measured by ultrasound was >12 mm. ET was then performed.

### Pregnancy Outcome Measures

The CP rate was determined by dividing the number of cycles or patients in which or whom a gestational sac was observed by ultrasound by the total number of cycles or patients in a certain group of patients. The miscarriage rate was calculated by dividing the total number of pregnancies by the number of pregnancies that did not show a fetal heartbeat on ultrasound or pregnancies in which the fetal heartbeat initially observed on ultrasound disappeared with symptoms associated with a miscarriage.

### Hormone Measurements

Serum anti-Müllerian hormone (AMH) concentrations in samples were measured in duplicate using the Anti-Mullerian Hormone Gen II enzyme linked immunosorbent assay kit (BECKMAN COULTER, Fullerton, CA, USA). The range of the assay was 0.14–21.0 ng/mL. The intra- and inter-assay coefficients for AMH determination were 12.3% and 14.2%, respectively. Serum FSH concentrations were measured by immune-enzymatic assay using ST AIA-PACK FSH (TOSOH AIA, Inc., Toyama, Japan). The range of the assay was 1.0–200 mIU/mL. The intra- and inter-assay coefficients were both <15%. Serum LH concentrations were measured by immune-enzymatic assay using ST AIA-PACK LH II (TOSOH AIA, Inc.). The range of the assay was 0.2–200 mIU/mL. The intra- and inter-assay coefficients were both <15%. Serum E2 concentrations were measured by enzyme immunoassay using ST AIA-PACK iE2 (TOSOH AIA, Inc.). The range of the assay was 20–3,000 pg/mL. The intra- and inter-assay coefficients were both <15%. Serum progesterone concentrations were measured by enzyme immunoassay using ST AIA-PACK PROG III (TOSOH AIA, Inc.). The range of the assay was 0.1–40 ng/mL. The intra- and inter-assay coefficients were both <15%.

### Statistical Analysis

Data were expressed as mean ± standard error (SE). Differences in rates and parametric data were compared using the chi-squared test, Mann–Whitney *U* test, or z-test. P-values <0.05 were considered statistically significant.

## Results

A total of 466 patients with POI who we first saw at our clinic between April 2014 and December 2017 were retrospectively analyzed until December 2020 under estrogen and progestin replacement therapy with or without repeated OS, as described above. OS was conducted in 6891 cycles in 429 patients (Group OS), whereas HR only (Group HR) was conducted in 1117 cycles in 37 patients. The overall CP and LB rates in these patients during the observation period were 14.2% (66/466) and 10.7% (50/466), respectively. In Group OS, follicle growth sufficient for OR or IUI/TI was obtained in 48.3% (207/429) of the patients and 15.1% (1043/6891) of the cycles, whereas in Group HR, the rates were 5.4% (2/37) and 0.2% (2/1117), respectively (p<0.01) (**Table 1**). When the patients in Group OS were classified into three groups by age at initiation of follow-up (a: <35, b: 35–39, c: ≥40 years), the follicle growth, CP, and LB rates per case in each group were as follows: Group a: 51.6% (81/157), 26.8% (42/157), and 21.0% (33/157); Group b: 49.7% (91/183), 12.0% (22/183), and 8.7% (16/183); and Group c: 39.3% (35/89), 2.2% (2/89), and 1.1% (1/89), respectively. The rate of follicle growth sufficient for attempting OR or IUI/TI per case was similar in all three age groups, but the CP and LB rates were significantly higher in Group a and decreased significantly in patients in the older age groups (p<0.01) (**Table 2**). Among the 429 patients in Group OS, 423 wished to have IVF-ET (Group IVF, n=423). In 62 patients in Group IVF who did not have indications of IVF-ET other than POI, IUI/TI was performed instead of OR at least once when desired because of financial or social circumstances. Six patients wished to have only IUI/TI from the start of follow-up. Thus, IUI/TI was performed in 236 cycles in 68 patients. We excluded three pregnancies resulting from IUI/TI from CP and LB rates in Group IVF. In Group IVF, OR was attempted in 807 cycles in 201 patients and 583 mature metaphase II (MII) oocytes were obtained. In cycles where IVF was attempted, the CP and LB rates per cycle, except for those in patients who wished to have oocyte cryopreservation (n=24, 49 cycles) and those who had cryopreserved embryos but had not undergone ET (n=13, 58 cycles), were 11.3% (79/700) and 6.9% (48/700), respectively, which were significantly higher than those in cycles where IUI/TI was attempted [1.3% (3/236) and 1.3% (3/236), respectively; p<0.01] (**Table 3**). No pregnancies occurred in Group HR.

**Table 1:** Follicle growth rate of patients in Groups OS and HR among patients with POI.

	Group OS	Group HR
Per patient	48.3% (207/429)**	5.4% (2/37)
Per cycle	15.1% (1043/6891)**	0.2% (2/1117)

Group OS: patients who underwent ovarian stimulation by hMG/recFSH with GnRH-a under hormone replacement; Group HR, patients who were observed with estrogen and progestin replacement only.

**p < 0.01.

**Table 2:** Follicle growth, clinical pregnancy, and live birth rates in Group OS classified by age at initiation of follow-up.

	Age Group		
	a (<35)	b (35–39)	c (≥40)
Follicle growth rate	51.6% (81/157)	49.7% (91/183)	39.3% (35/89)
Clinical pregnancy rate	26.8% (42/157)**	12.0% (22/183)	2.2% (2/89)
Live birth rate	21.0% (33/157)**	8.7% (16/183)	1.1% (1/89)
Abortion rate	21.4% (9/42)	27.3% (6/22)	50.0% (1/2)

Group OS, patients who underwent ovarian stimulation by hMG/recFSH with GnRH-a under hormone replacement; Group HR, patients who were observed with estrogen and progestin replacement only.

a, patients <35 years; b, patients 35–39 years; c, patients ≥40 years of age at initiation of follow-up at Rose Ladies Clinic.

**p < 0.01.

**Table 3:** Clinical pregnancy and live birth rates in cycles in which either IVF or IUI/TI was attempted in patients in Group OS.

	IVF cycle	IUI/TI
Clinical pregnancy rate	11.3% (79/700)**	1.3% (3/236)
Live birth rate	6.9% (48/700)**	1.3% (3/236)

IVF, in vitro fertilization; IUI, intrauterine insemination; TI, timed intercourse.

Group OS, patients who underwent ovarian stimulation by hMG/recFSH with GnRH-a under hormone replacement.

Cycles in patients who wished to have oocyte cryopreservation (n=24, 49 cycles) and in patients who had cryopreserved embryos but had not undergone ET (n=13, 58 cycles) were excluded.

**p < 0.01.

**Figure 1:**
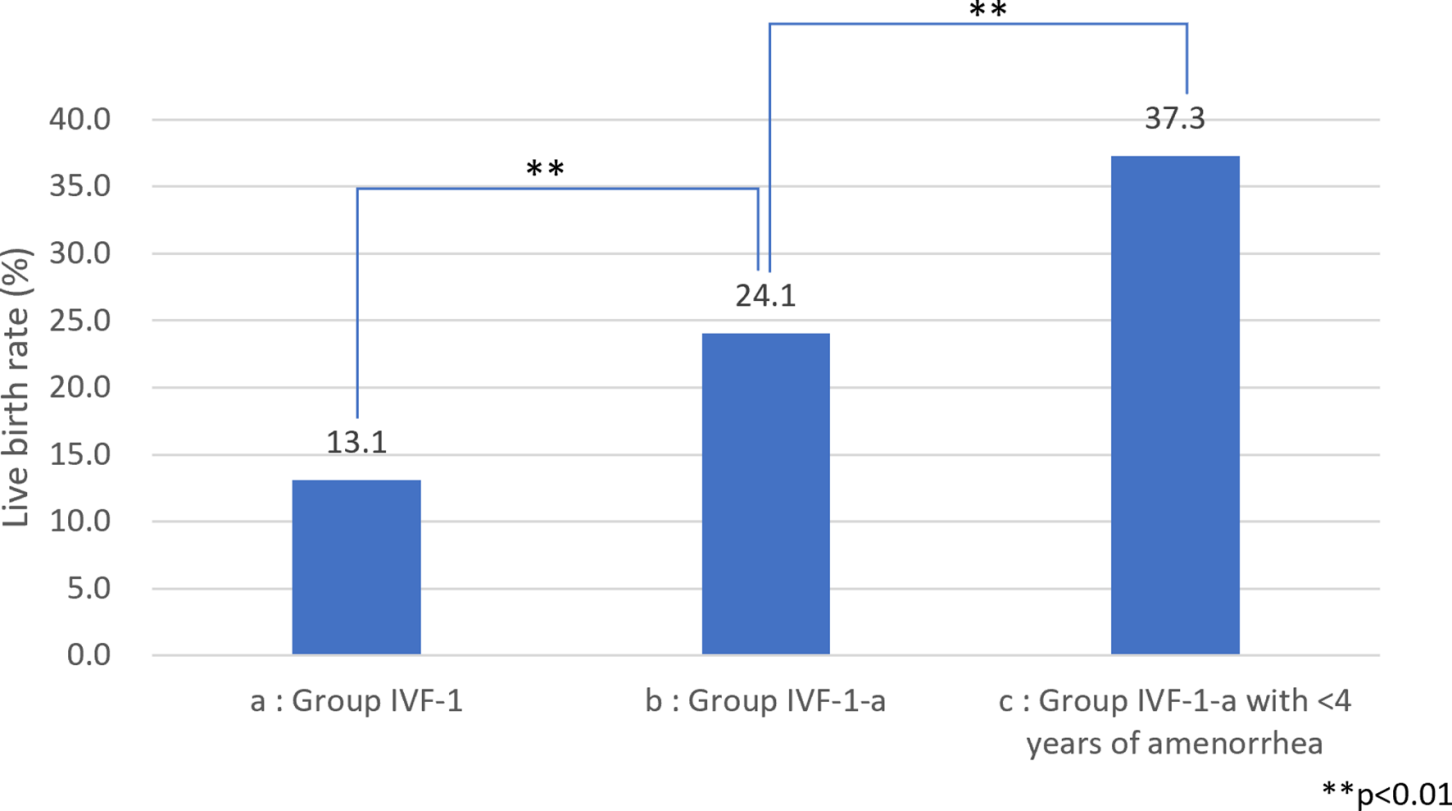
Live birth (LB) rate among patients with idiopathic premature ovarian insufficiency (POI) **(A)** LB rate among patients with idiopathic POI who underwent ovarian stimulation with human menopausal gonadotropin (hMG)/recombinant follicle-stimulating hormone (recFSH) with GnRH-a under hormone replacement (OS) and *in vitro* fertilization (IVF-ET). **(B)** LB rate among patients with idiopathic POI who underwent OS and IVF-ET aged <35 years at the start of follow-up. **(C)** LB rate among patients with idiopathic POI who underwent OS and IVF-ET aged <35 years at the start of follow-up with <4 years of amenorrhea.

Among the 583 MII oocytes retrieved, 33 in 24 patients were cryopreserved for future IVF. IVF/ICSI was attempted with 550 oocytes. The overall embryo cryopreservation rate was 59.3% (326/550). The average number of OS cycles in Group IVF was 9.2 ± 0.3 (range, 1–34). When the patients in Group IVF were classified into three groups according to the age at which they started follow-up, namely, a, b, and c, as described above, the CP and LB rates were significantly higher in Group IVF-a [28.9% (39/136) and 22.0% (30/136)] than in Group IVF-b [13.3% (22/165) and 9.7% (16/165)] or Group IVF-c [2.4% (2/85) and 1.2% (1/85)], respectively (p<0.01) (**Table 4**). The patients in Group IVF (n=423), except for those who wished to have oocyte cryopreservation (n=24) and those who obtained embryos by IVF but still had not attempted ET by December 2020 (n=13), were divided into four groups based on possible etiological factors, as described above: 297 patients in Group IVF-1, 50 in Group IVF-2, 17 in Group IVF-3, and 22 in Group IVF-4. The CP and LB rates in the patients in these four groups were as follows: Group IVF-1, 17.8% (53/297) and 13.1% (39/297); Group IVF-2, 16.0% (8/50) and 14.0% (7/50); Group IVF-3, 0% and 0%; and Group IVF-4, 9.1% (2/22) and 4.5% (1/22), respectively. Among the 47 LBs among the patients who underwent IVF-ET, 39 were in Group IVF-1, 7 in Group IVF-2, none in Group IVF-3, and only 1 in Group IVF-4 (**Table 5**). One patient in Group IVF-1 achieved two LBs during follow-up. When we divided the patients in Group IVF-1 (idiopathic POI) who had completed IVF-ET (n=297) into three age groups as described above (a: <35, b: 35–39, c: ≥40 years), the CP and LB rates in Group IVF-1-a were 31.5% (34/108) and 24.1.4% (26/108), respectively, and decreased significantly in patients who were aged ≥35 years at the initiation of follow-up (**Table 6**). Furthermore, the CP and LB rates per case in the patients in Group IVF-1-a with amenorrhea for <4 years at the first visit to our facility were 49.0% (25/51) and 37.3% (19/51), respectively, and decreased significantly as the duration of amenorrhea at the first visit to our facility increased to 4–8, 8–11, and ≥12 years. Two patients with 8–11 years of amenorrhea achieved an LB, but no pregnancies occurred in patients with ≥12 years of amenorrhea at the initiation of follow-up (**Table 7**). **Figure 1** shows the LB rates in Groups IVF-1 and IVF-1-a, and in patients in Group IVF-1-a with <4 years of amenorrhea (p<0.01). The CP and LB rates per case in patients in Group IVF-2 with <4 years of amenorrhea at the initiation of follow-up were 24.2% (8/33) and 21.2% (7/33), respectively. No pregnancies occurred in patients in Group IVF-2 with ≥4 years of amenorrhea (**Table 8**). Patient No. 4 achieved two LBs during the follow-up period. A list of patients who achieved an LB is shown in **Table 9**. The mean ± SE duration of amenorrhea at the initiation of follow-up of those 50 patients was 2.8 ± 0.4 (median, 1.7; range, 0.3–9.2). Among these 50 patients, the patient with the longest duration of amenorrhea at the initiation of follow-up was the one in Group 1 with 9.2 years. The mean number of OS cycles until the embryos which achieved pregnancies were obtained or conception by IUI/TI was 4.4 ± 0.4 (median, 3.5; range, 1.0–15.0). The mean ± SE duration of amenorrhea in patients who eventually achieved an LB in Group 1 was significantly longer than that in patients in Group 2 (3.0 ± 0.4 *vs.* 1.2 ± 0.5, respectively; p<0.05). A patient with a chromosomal abnormality who carried a Robertsonian translocation [45, XX,der (4,21) (q10;q10)] eventually achieved an LB after obtaining five frozen embryos and conceiving at the third ET.

**Table 4:** Clinical pregnancy and live birth rates per case in patients in Group IVF.

	Age Group		
	a (<35)	b (35–39)	c (≥40)
Clinical pregnancy rate	28.9% (39/136)**	13.3% (22/165)	2.4% (2/85)
Live birth rate	22.0% (30/136)**	9.7% (16/165)	1.2% (1/85)

Group IVF, patients in whom ovarian stimulation and IVF-ET were attempted.

a, patients <35 years; b, patients 35–39 years; c, patients ≥40 years of age at initiation of follow-up.

Patients who wished to cryopreserve oocytes (n=24) and patients who had cryopreserved embryos but had not undergone embryo transfer (n=13) were excluded.

**p < 0.01.

**Table 5:** Clinical pregnancy and live birth rates per case in patients in Group IVF categorized into four groups by possible etiological factors.

	Patients with POI Classified by Etiological Factors			
	Group IVF-1 (Idiopathic)	Group IVF-2 (Iatrogenic-1)	Group IVF-3 (Iatrogenic-2)	Group IVF-4 (Chromosomal abnormality)
Clinical pregnancy rate	17.8% (53/297)	16.0% (8/50)	0.0% (0/17)	9.1% (2/22)
Live birth rate	13.1% (39/297)	14.0% (7/50)	0.0% (0/17)	4.5% (1/22)

Group IVF, patients in whom ovarian stimulation and IVF-ET were attempted; Group IVF-1 (idiopathic), patients without possible iatrogenic causes or chromosomal abnormalities; Group IVF-2 (iatrogenic-1), patients with past surgery for benign ovarian tumors; Group IVF-3 (iatrogenic-2), patients with past treatment for malignancy; Group IVF-4 (chromosomal abnormality), patients with chromosomal abnormalities.

**Table 6:** Clinical pregnancy and live birth rates in patients in Group IVF-1 classified into three groups by age at initiation of follow-up.

	Age Groups in Patients in Group IVF-1		
	a (<35)	b (35–39)	c (≥40)
Clinical pregnancy rate	31.5% (34/108)**	14.2% (18/127)	1.6% (1/62)
Live birth rate	24.1% (26/108)**	10.2% (13/127)	0.0% (0/62)
Abortion rate	23.5% (8/34)	27.8% (5/18)	100.0% (1/1)

Group IVF-1, patients without possible iatrogenic causes or chromosomal abnormalities in whom ovarian stimulation and IVF-ET were attempted.

a, patients <35 years of age at the initiation of follow-up; b, patients 35–39 years of age at the initiation of follow-up; c, patients ≥40 years of age at the initiation of follow-up.

**p < 0.01.

**Table 7:** Clinical pregnancy and live birth rates in patients in Group IVF-1-a with different durations of amenorrhea at the initiation of follow-up.

	Duration of Amenorrhea (years)			
	<4	4–7	8–11	≥12
Clinical pregnancy rate	49.0% (25/51)*	24.1% (7/29)	11.1% (2/18)	0.0% (0/10)
Live birth rate	37.3% (19/51)	17.2% (5/29)	11.1% (2/18)	0.0% (0/10)

Group IVF-1-a, patients without possible iatrogenic causes or chromosomal abnormalities in whom ovarian stimulation and IVF-ET were attempted abnormality and were <35 years of age at the initiation of follow-up.

*p < 0.05.

**Table 8:** Clinical pregnancy and live birth rates in patients in Group IVF-2 with different durations of amenorrhea at the initiation of follow-up.

	Duration of Amenorrhea (years)	
	<4	≥4
Clinical pregnancy rate	24.2% (8/33)	0.0% (0/12)
Live birth rate	21.2% (7/33)	0.0% (0/12)

Group IVF-2, patients with a history of surgery for benign ovarian tumors in whom ovarian stimulation and IVF-ET were attempted.

**Table 9:** List of patients.

No.	Age at the Start of Follow-Up (Years)	Group	Duration of Amenorrhea at the Start of Follow-Up (Years)	Mode of Conception	Number of OS Cycles Until Obtaining Embryos that Led to a Live Birth Were Cryopreserved	Period from the Start of Follow-Up to the Cryopreservation of Embryos Leading to a Live Birth (Years)
1	23	1	0.3	IUI	3^#^	0.5
2	26	1	7.0	IVF-ET	6	0.7
3	27	1	8.7	IVF-ET	1	0.1
4	28	1	0.8	IVF-ET	2	0.3
				IVF-ET	10	4.5
5	29	1	2.1	IVF-ET	1	0.1
6	29	1	1.1	IVF-ET	8	1.5
7	30	1	0.4	IVF-ET	11	2.4
8	30	1	0.7	IVF-ET	3, 4	0.5, 0.6
9	30	1	1.3	IVF-ET	7	1.4
10	30	1	2.0	IVF-ET	7	1.3
11	30	1	1.1	IVF-ET	5	0.8
12	31	1	7.5	IVF-ET	6	1.8
13	31	1	2.8	IVF-ET	4	0.5
14	31	1	3.3	IVF-ET	2, 5	0.4, 1.0
15	31	1	0.4	IVF-ET	2	1.1
16	32	1	8.5	TI	8^#^	3.0
17	32	1	3.9	IUI	3^#^	0.5
18	32	1	1.6	IVF-ET	2	0.5
19	32	1	6.5	IVF-ET	6	1.0
20	32	1	7.1	IVF-ET	2	0.1
21	32	1	1.2	IVF-ET	5	0.8
22	33	1	1.3	IVF-ET	7	1.5
23	33	1	1.5	IVF-ET	3, 4	0.6, 0.7
24	34	1	9.2	IVF-ET	5, 10	2.3, 3.2
25	34	1	0.3	IVF-ET	5	1.9
26	34	1	5.0	IVF-ET	2, 9	0.1, 1.5
27	34	1	1.4	IVF-ET	4	0.9
28	34	1	1.1	IVF-ET	1, 2	0.1, 0.3
29	34	1	3.1	IVF-ET	3	1.1
30	35	1	5.5	IVF-ET	1	0.8
31	35	1	1.2	IVF-ET	1	0.2
32	36	1	3.0	IVF-ET	2	0.3
33	36	1	0.3	IVF-ET	2	0.5
34	36	1	3.3	IVF-ET	1	0.3
35	37	1	6.0	IVF-ET	3	1.0
36	37	1	3.7	IVF-ET	5	0.7
37	37	1	4.8	IVF-ET	2	0.5
38	37	1	0.5	IVF-ET	2	0.5
39	38	1	1.8	IVF-ET	4	0.9
40	38	1	0.8	IVF-ET	1, 2	0.3, 0.4
41	39	1	3.5	IVF-ET	4, 10	0.5, 1.6
42	39	1	0.8	IVF-ET	8	1.3
43	31	2	0.6	IVF-ET	2	0.3
44	34	2	2.0	IVF-ET	3	3.0
45	34	2	0.6	IVF-ET	5	0.6
46	37	2	0.3	IVF-ET	2, 15	1.1, 2.1
47	37	2	0.3	IVF-ET	3	1.1
48	38	2	0.9	IVF-ET	1	0.2
49	40	2	3.6	IVF-ET	8	1.3
50	32	4	3.3	IVF-ET	6	1.5
Median (min-max)	33.5 (23.0–40.0)	–	1.7 (0.3–9.2)	–	3.5 (1.0–15.0)	0.8 (0.1–4.5)
Mean ± SE	33.2 ± 0.5	–	2.8 ± 0.4	–	4.4 ± 0.4	1.0 ± 0.1

OS, ovarian stimulation; IUI, intrauterine insemination; TI, timed intercourse.

Group 1, patients without possible iatrogenic causes or chromosomal abnormalities; Group 2, patients with past surgeries for benign ovarian tumors; Group 4, patients with chromosomal abnormalities.

^#^Number of OS cycles until becoming pregnant via IUI or TI.

**Figure 2:**
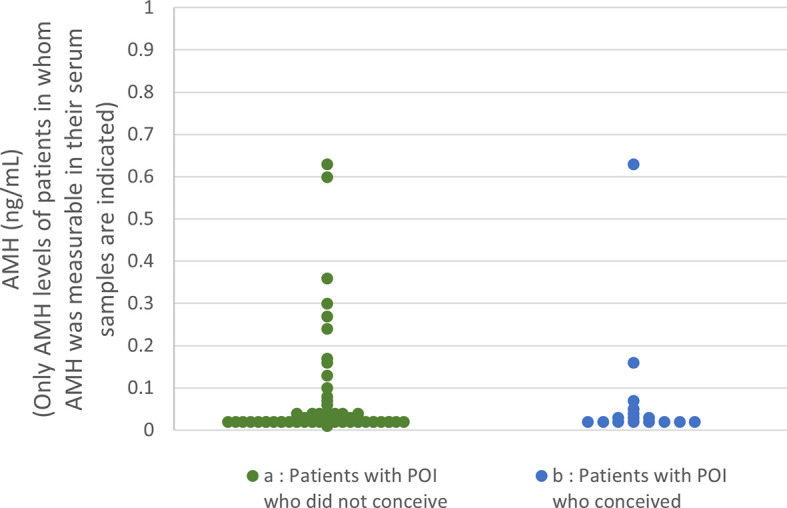
Serum anti-Müllerian hormone (AMH) distribution among the patients in the present study **(A)** In patients who did not conceive (74.5% of the patients’ levels were below the detection limit), only the levels of samples in which AMH was measurable are plotted. **(B)** In patients who achieved a live birth (70.7% of the patients’ levels were below the detection limit), only the levels of samples in which AMH was measurable are plotted.

Serum AMH levels at the initiation of follow-up among the patients in the present study are shown in **Figure 2**. AMH levels were below the detectable limit of the assay in 70.7% of the patients who conceived and 74.5% of the patients who did not (n.s.). No significant difference was found in AMH levels between the two groups.

## Discussion

In their mixed retrospective and prospective study on 358 patients with POI, Bidet et al. reported that spontaneous remission occurred in 24% of the cases (4). Our data showed that 48.3% of the cases in whom OS was repeated showed follicle growth sufficient for OR or IUI/TI, while 5.4% of the cases followed only with HR showed follicle growth by the same definition. Follicle growth was observed in 15.1% of all cycles in which OS was performed, compared with only 0.2% of the cycles in patients followed with HR only. This finding suggested that OS under HR is effective for stimulating follicle growth in at least some patients with POI.

No pregnancies occurred in patients who were followed with HR only. The combined spontaneous pregnancy rate per patient from the six studies cited in the review by van Kasteren et al. (5) was 4.8% among 558 patients (27/558) who were observed for 0.5–10 years only with HR (16–21). In addition, there has been report of spontaneous pregnancy in patients with POI after 10 years of amenorrhea (22). The pace of ovarian reserve decline may vary among patients with POI depending on the etiology and genetic differences (23–25). Therefore, the spontaneous pregnancy rate may have increased if we had followed the patients for a longer period, even those with only HR.

Infertility treatment is generally considered to be ineffective in patients with POI. Among the reports on pregnancies in patients with POI, most were under estrogen replacement, suggesting a positive effect of estrogen on follicle growth by lowering gonadotropin levels through a negative feedback loop (26–35). Furthermore, in studies of attempted ovulation induction in patients with POI, successful ovulation and pregnancy by hMG stimulation were obtained mainly in cases under estrogen replacement (10, 11, 36–39). In a study in which ovulation induction was attempted by hMG combined with GnRH-a without estrogen replacement, the pregnancy rates were 0% and 3.4% in patients with >3 months or >6 months of amenorrhea, respectively (36, 40).

Taken together, these results suggest that follicle growth was induced in patients in whom gonadotropin levels were suppressed under certain threshold levels with estrogen replacement, which has also been shown to enhance FSH signaling in granulosa cells. This hypothesis was supported by animal studies that demonstrated that E2 enhances FSH binding to its receptors (41, 42). GnRH-a administration alone was reported to cause sporadic spontaneous ovulation in a patient with POI (43, 44). GnRH-a administration may also prevent the premature LH surge that interferes with full follicle maturation. Premature luteinization may be induced by decreased negative feedback as a consequence of poor follicle reserve and increased LH levels in patients with POI attempting ovulation induction (45). In the present study, we attempted to suppress serum LH levels to the normal range during the early follicular phase of the ovulatory cycle by controlling estrogen and GnRH-a dosages during OS to prevent sporadic LH spikes while raising FSH levels >30 mIU/mL by exogenous gonadotropins.

However, to our knowledge, no large-scale cohort studies reporting LB rates in patients with POI who wish to conceive using their own eggs have been conducted. In the present study, we investigated the LB rate in patients with POI during long-term follow-up under HR therapy with or without OS. The ESHRE guidelines cite the reports by Tartagni et al. (11) and Badawy et al. (40) and state that further randomized controlled trials are needed to confirm the potential beneficial effects of gonadotrophin suppression (using either estrogen or GnRH-a), with pregnancy as the main outcome measure. In the present large-scale retrospective analysis, we partially revealed the effects of gonadotropin suppression with GnRH-a under HR with hMG stimulation on the live birth rate in some patients with POI. There were 51 LBs in 50 patients during the follow-up period. All of the LBs occurred in cases in whom ovulation induction was attempted by hMG/recFSH with GnRH-a. In previous studies in which ovulation induction was attempted by recFSH/hMG with GnRH-a, the duration of administration was either 10 days, 3 weeks, or 10 days, and repeated four times at most (7, 10, 40). In the present study, we performed recFSH/hMG stimulation (225–375 IU/day) with GnRH-a under estrogen replacement continuously for 4 weeks, even if no follicle growth was observed in cycles that were repeated as consecutively as possible if circumstances allowed. Because it takes >3 months for secondary or early antral follicles to grow to the preovulatory stage, the findings of the present study suggest that OS under HR continues to support follicle growth through 4-week cycles until being sufficient for IVF-ET or IUI/TI (46, 47). Thus, OS may be beneficial for infertility treatment in patients with POI by supporting follicle growth over a longer duration than that reported in previous studies (10, 36).

In the present study, although the reason for this difference was unclear, the LB rate was significantly higher in cycles in which IVF-ET was attempted than in cycles in which IUI/TI was attempted. We employed a “freeze-all” strategy and transferred embryos in later cycles when the endometrial condition was optimized by exogenous E2 administration and the serum E2 levels were increased to >350 pg/mL, which may have contributed to the higher CL and LB rates in the IVF cycles. We conducted a “freeze-all” strategy to accumulate as many embryos as possible before initiating ET because the ovarian reserve of patients with POI may continue to decrease, even during the follow-up period, and the duration of potential follicle growth by OS could be limited. The timing of starting ET was determined by considering the response to OS, patient age, and the number of cryopreserved embryos in each patient.

In many cases of POI in whom we attempted IVF-ET in the past, extending culture to the blastocyst stage resulted in attrition of embryos that otherwise might support viable pregnancies following cleavage-stage transfer (48). We chose to freeze all embryos at the four-to-six-cell stage for future ET to E2-primed endometrium. Thus, by utilizing IVF-ET, we aimed to increase the LB rate in patients with POI.

In the present study, we demonstrated for the first time an age difference in CP and LB rates in response to infertility treatment in patients with POI (5, 49). In the patients in Group OS, the overall CL and LB rates were higher in those aged <35 years at the initiation of follow-up and deteriorated in patients who started the follow-up at age ≥35 years, while the follicle growth rate did not significantly differ among the three age groups. Thus, independent of the ovarian reserve, which is diminished by etiological factors, the quality of oocytes deteriorates as patients with POI age (**Table 2**). It is presumed that the quality of oocytes is generally preserved in patients with POI.

We also demonstrated in the present study that, among the patients with POI with different etiological factors, the patients in Groups 1 and 2 had a greater chance of responding to OS (**Table 5**). It is understandable that the patients in Groups 3 (after treatment for malignancy) and 4 (chromosomal abnormalities) suffered heavier damage to their ovarian reserves. We demonstrated that patients in Group IVF-1 achieved an LB at the rate of 13.1% (39/297) per patient, which was higher than that of 4.2% (15/358) among 358 patients with idiopathic POI without OS reported by Bidet et al. (4) (p<0.01) (**Table 5**). We further confirmed that the LB rate increased to 24.1% (26/108, p<0.01) in patients in Group IVF-1-a and to 37.3% in patients in Group IVF-1-a who had a duration of amenorrhea <4 years (19/51, p<0.01) (Fig. 1). It is presumed that most of the patients in Group 1 had a genetic basis as an etiology, which caused ovarian dysfunction at various levels of severity with a variety of time courses. There are about 50 genes in which mutations are known to be causative of POI. These genes may account for only a small proportion of patients, and the majority of such patients do not have a genetic diagnosis (23, 50). Interestingly, in the present study, some patients in Group IVF-1 with ≥8 years of amenorrhea underwent OR, and some even achieved an LB. These patients may have certain genetic characteristics that may be predictable by genetic diagnosis in the future. Improved understanding of the genetic causes of POI should lead to better indications for prognosis and fertility potential in patients with POI (23).

The relatively high abortion rates of 21.4% even among the patients in our study aged <35 years and 23.5% (8/34) among those in Group IVF-1-a are in accordance with the rate of about 20% reported in the systematic review by van Kasteren et al. (5); this result should be further confirmed in a future study (**Tables 2**, **6**).

It has been reported that 10.0%–12.5% of patients with POI have chromosomal abnormalities (25, 51–54). Most of the chromosomal abnormalities in patients with POI are aneuploidy or structural abnormalities involving the X chromosome (55). Robertsonian translocation occurs at the rate of 1/1000 and is known to be associated with an increased prevalence of miscarriage, but ovarian function has been thought to be preserved. We and another group have previously reported data suggesting an increased prevalence of this chromosomal abnormality among patients with POI (25, 51). As suggested in the previous data, Robertsonian translocation may present with different degrees of ovarian dysfunction besides recurrent pregnancy loss, as previously suggested (56).

Our present result that serum AMH measurement did not predict the LB rate by OS in patients with POI as accurately as etiological factors, age, and duration of amenorrhea at initiation of follow-up, at least with the assay we used, is not in accordance with the predictability of AMH values in terms of the number of residual follicles by histological examination in patients with idiopathic POI reported by Meduri et al. (57). Although the reason for this discrepancy is unclear, some cases in their report had AMH levels >1 ng/mL; no such cases were observed in the present cohort. In addition, they did not mention the duration of amenorrhea among their patients. Therefore, this difference may be explained by differences in the patient population, or, as they mentioned in their report, by defects in antral follicle development in patients with POI, even those with measurable serum AMH levels (57).

The present data also revealed that 48.3% of our patients with POI showed follicle maturation sufficient for OR or IUI/TI (**Table 1**). Thus, these patients may retain a certain follicle reserve size to respond to OS, which suggests that the recently developed *in vitro* activation of primordial follicles may facilitate further follicle growth in such patients. We are currently conducting a clinical trial utilizing this approach (58–60).

With the development of genetic diagnoses and more sensitive markers of preantral/small antral preantral follicles, OS as described in this study may be performed more effectively and inexpensively in the future.

## Conclusion

The results of this study suggest that infertility treatment is possible in some patients with POI, especially in patients in Groups IVF-1-a and 2 with <4 years of amenorrhea. In such patients, OS under HR should be considered before attempting OD.

## Data Availability Statement

The original contributions presented in the study are included in the article/supplementary material. Further inquiries can be directed to the corresponding author.

## Ethics Statement

The studies involving human participants were reviewed and approved by Biomedical Ethics Committee of the Rose Ladies Clinic (RLC-19) and registered in the University Hospital Medical Association Network (UMIN 000040360). Written informed consent for participation was not required for this study in accordance with the national legislation and the institutional requirements.

## Author Contributions

BI was the principal investigator and led the clinical team. MF, MK, and KK pursued the clinical work. EK was the study coordinator and was involved in the statistical aspects of the study. KK was also involved in designing the study. All authors contributed to the article and approved the submitted version.

## Conflict of Interest

The authors declare that the research was conducted in the absence of any commercial or financial relationships that could be construed as a potential conflict of interest.

## Publisher’s Note

All claims expressed in this article are solely those of the authors and do not necessarily represent those of their affiliated organizations, or those of the publisher, the editors and the reviewers. Any product that may be evaluated in this article, or claim that may be made by its manufacturer, is not guaranteed or endorsed by the publisher.
